# Adolescent Cranial Volume as a Sensitive Marker of Parental Investment: The Role of Non-material Resources?

**DOI:** 10.3389/fpsyg.2020.602401

**Published:** 2020-12-15

**Authors:** Velda Lauringson, Gudrun Veldre, Peeter Hõrak

**Affiliations:** ^1^Department of Zoology, University of Tartu, Tartu, Estonia; ^2^Department of Anatomy, Centre for Physical Anthropology, University of Tartu, Tartu, Estonia

**Keywords:** cranial volume, family structure, height, meat shortage, parental investment, paternal education

## Abstract

Growth of different body parts in humans is sensitive to different resource constraints that are mediated by parental investment. Parental investment can involve the expenditure of material, cognitive, and emotional resources on offspring. Cranial volume, an important predictor of cognitive ability, appears understudied in this context. We asked (1) whether there are associations between growth and family structure, self-reported estimates for resource availability, and sibling number; and (2) whether these constraints relate to head and body growth in a similar manner. We assessed the associations between parental investment, height, and cranial volume in a cross-sectional study of Estonian children (born 1980–87, aged 11–17). Height correlated negatively with the number of siblings but this association became negligible in a model controlling for birthweight, parental heights, and mother’s age at birth. Unlike height, cranial volume was unrelated to sibling number, but it was negatively associated with self-reported meat and general resource shortage. Cranial volume was related to family structure and paternal education. Children living with both birth-parents had larger heads than those living in families containing a step-parent. Since these family types did not differ with respect to meat or general resource shortage, our findings suggest that families including both genetic parents provide non-material benefits that stimulate predominantly cranial growth. For the studied developmental period, cranial volume appeared a more sensitive marker of growth constraints than height. The potential of using cranial volume for quantifying physical impact of non-material parental investment deserves further attention.

## Introduction

Growth and development of organisms are constrained by physiological and microevolutionary trade-offs that result from allocation of limited amount of resources between different components of fitness (Stearns, 1992). For example, the quality of offspring is traded off against offspring number in modern human populations of low fertility and mortality (reviewed in Lawson and Mace, 2010). Physiological trade-offs can be defined as alteration in allocation of resources between different traits or functions in response to externally or internally induced changes. An example of a physiological trade-off is growth stunting in response to infections that require allocating somatic investments into immune responses at the expense of growth (McDade, 2003; Hõrak and Valge, 2015) or sacrificing the growth of some organs or tissues (such as viscera) to protect other organs whose function would be more detrimentally influenced by impaired growth (e.g., brain) in response to intra-uterine nutrient limitation (“thrifty phenotype,” Hales and Barker, 1992; Wells, 2013). Microevolutionary trade-offs reflect within-population (or higher-order) genetic diversity in continua of life-history and physiological strategies (see Hõrak and Cohen, 2010). An extreme example of a microevolutionary trade-off is a human pygmy phenotype that results from early growth cessation that has supposedly evolved to facilitate early reproductive onset under conditions of high adult mortality (Migliano et al., 2007). Microevolutionary trade-offs also manifest within modern populations as genetic correlations between life-history, physiological, and behavioral traits (see e.g., Day et al., 2016).

Variation in the amount of resources that are available for individuals can alter the sign of correlations between life-history traits (van Noordwijk and de Jong, 1986): When among-individual variation in resource acquisition is smaller than variation in resource allocation, negative correlations (i.e., the trade-offs) between the components of fitness can be observed. When relative variation in resource acquisition is larger than variation in resource allocation among individuals, positive correlations between life-history traits can be observed. For instance, in several traditional societies, parents with more children are also able to invest more into each child, so that a positive relationship between the number of siblings and their quality (measured as childhood survival) emerges (Walker et al., 2008; Lawson and Mace, 2010).

Growth of different body parts in humans is subject to different constraints (Wadsworth et al., 2002; Hõrak and Valge, 2015), not least because of trade-offs in resource allocation between the growth of different organ systems (reviewed in Said-Mohamed et al., 2018). For instance, the brain–body growth trade-off hypothesis (Kuzawa et al., 2014) proposes that high costs of human brain development require compensatory slowing of body growth rate at a time when the energy demand of a growing brain peaks. Consequently, one might predict that body and head growth are subject to different constraints and/or optimization rules (Wells, 2013).

Trade-offs in human growth are mediated by parental investment. Parents expend material as well as cognitive and emotional resources to benefit current offspring at the cost to their ability to invest in other components of fitness. This resource limitation provides an arena for parent-offspring, inter-offspring, and inter-parental conflicts and cooperation that results from antagonistic (yet partially overlapping) genetic interests of siblings, their parents, and partners of the parents (Trivers, 1972; Daly and Wilson, 1980). Such conflicts are most prominent in families including step-parents (particularly step-fathers) who provide less direct care, monetary support, financial aid for continued education, playtime, and homework help to their stepchildren than do biological parents (reviewed in Anderson, 2000; Hamilton et al., 2007).

So far only a few studies have connected living with step-parents with suboptimal linear growth of children (Flinn et al., 1999; Li et al., 2004; Mace, 2015); yet to our knowledge, the question about whether or how family structure relates to offspring brain growth or development has remained unanswered. There are, however, reasons to expect that human brain is particularly sensitive to parental investment. Several studies have documented secular increases in the size of various cranial measures from the mid-nineteenth or the early twentieth century (reviewed in Woodley of Menie et al., 2016; Kim et al., 2018) that parallel secular increases in height (e.g., Hõrak and Valge, 2015). Severe undernutrition during infancy inhibits brain growth and subsequent intellectual development among disadvantaged children (reviewed in Ivanovic, 1996). However, availability of essential micronutrients may constrain brain growth and development even in adequately nourished populations (e.g., Caballero, 2002; Arija et al., 2006). For instance, dietary scarcity of long-chain polyunsaturated fatty acids (that make up 20% of the dry weight of human brain) may negatively affect cognitive ability (Lassek and Gaulin (2008, 2015). Further, natural variation in parental (Kok et al., 2015) or maternal (Luby et al., 2012; Whittle et al., 2016) support in early childhood has been shown to predict later child structural brain development in contemporary western societies.

Understanding how external constraints via parental investment relate to head and body growth is important because both stature and cranial volume (a proxy for brain size) correlate phenotypically and genetically with cognitive abilities and/or educational attainment (Nave et al., 2019; Valge et al., 2019; Jansen et al., 2020). Intelligence and education, in turn, strongly and robustly predict essential life outcomes including occupational status, happiness, health, and life expectancy (Deary et al., 2019).

The aim of this study is to assess the relationship between parental investment and height and cranial volume of Estonian schoolchildren (born 1980–87) from the city of Tartu who were measured around the 14 years of age. Specifically, we ask (1) whether there are associations between growth, family structure (i.e., single parent vs. two birth-parents vs. birth-parent + step-parent), self-reported estimates of resource availability, and sibling number; and (2) whether these constraints relate to the growth of head and body in a similar manner. In addition, we asked whether different growth constraints affect boys and girls in a similar manner in the light of an idea that males are less buffered against the environmental effects on growth and development (Stinson, 1985). For instance, studies in Latvia in the period following economic transition at the end of twentieth century have reported that material resource shortage at infancy relates to the height of men more strongly than to the height of women (Krams et al., 2019; Rubika et al., 2020).

Our setting is particularly suitable for the examination of growth constraints, as the pubertal growth spurt (which is extremely sensitive to external constraints) of most of the respondents coincided with the period of rapid transition to market economy in the 1990s, resulting in dramatic decline in real wages and rapid and massive socioeconomic stratification in Estonian society (Kaasik et al., 1998; Koupilova et al., 2000). This natural experiment provides a unique opportunity to study somatic growth in the context of highly variable resource availability in an otherwise homogenous setting within a single city and a short time span of data collection. Availability of data of birth weights, parental heights, and education enables statistical control for important genetic and/or biosocial confounding and mediating variables affecting growth.

## Materials and Methods

### The Dataset

Data on morphometric measurements and family background were obtained from an anthropometric study performed between 1997 and 1999 (Veldre et al., 2001, 2002). The cross-sectional sample consisted of 822 randomly selected adolescents from different schools of Tartu (about 100,000 inhabitants), Estonia. The dataset involved 418 girls and 404 boys (with average age of 13.8, *SD* = 1.2, range = 10.9–17.2 years). Birth years of the subjects ranged from 1980–87 and all of them were of Estonian origin. Parents and children themselves gave their consent to the voluntary examination; the study was approved by the Medical Ethics Committee of the University of Tartu. Retrospective data analysis for the current study was carried out anonymously under the license of the Research Ethics Committee of the University of Tartu (protocol # 297/T-2, issued on 21.10.2019).

### Anthropometric Measures

Stature was measured on mornings at school with a precision of 1 mm. Head length (the distance between *glabella* and *opisthocranium*) and width (the distance between both *euryon* points) were measured with Campbell sliding caliper (Rosscraft Centurion Kit; Rosscraft, Surrey, BC, Canada). Cranial volume was calculated according to Rushton (1997): 7.884^∗^(head length-11)+(10.842^∗^head width-11)-1593.96 for girls and 6.752^∗^(head length-11)+(11.421^∗^head width-11)-1434.06 for boys (units in mm). Since morphometric traits change with age, we used the raw data described above to calculate age-specific residuals for height and cranial volume. Residuals were obtained from generalized additive models in which the focal trait was regressed against smooth non-parametric functions of age (in days) using the package “gam” for R (Hastie, 2018); R syntax: focal trait ∼s(age). Residuals were then standardized to *z*-scores within sexes.

### Questionnaire

Children together with their parent(s) filled a questionnaire requiring information about their birth weight, education and height of parents, mother’s age at the birth of the subject, number of siblings (including step-siblings), and family structure (currently living with single parent, one birth-parent+step-parent, or two birth-parents). Coding of the family structure is specified in the headings of Tables 1, 2. Variation in maternal education was too low for meaningful analysis as only 28 mothers had primary education. We thus used only data for paternal education which was coded as binary factor: 1 if the father had primary education (8 years of schooling or less, *n* = 60) and 0 if his education was secondary or tertiary (*n* = 542). Use of binary classification for paternal education was based on the rationale that not obtaining higher than primary education could be considered as a handicap for the studied generation, while not obtaining tertiary education was not a handicap. This is because since the second half of 1960s, universal secondary education was proclaimed the goal in Estonia and the transition to compulsory secondary education was completed by 1980 (Saar, 2008). During the economic transition in the 1990s, persons with only primary education suffered disproportionately high losses in income and increases in unemployment rates (Noorkõiv et al., 1998). Two specific questions were relevant to growth constraints. The first question concerned the shortage of certain foods in children’s current diet: “My menu is short in … (name the food products).” From this item we extracted four binary factors termed meat shortage, milk shortage, fruit shortage, and sweets shortage which were assigned a value of 1 if the subject had reported that his/her menu is short in a specific food item (0 denotes cases where shortage was not reported, not missing data, as only questionnaires where the question about menu was answered were accounted for). Binary associations between food item shortages and metric variables are presented in Table 1 in case of meat and in Supplementary Table S1 in the Electronic Supplementary Material (ESM) for milk, fruit, and sweets shortage. Fruit and sweets shortages were not associated with children’s height and cranial volume in the binary analyses while children reporting milk shortage were on average shorter than those not reporting milk shortage (Supplementary Table S1). This association, however, faded in an ANCOVA model accounting for mediators and confounders [Table 2A; *F*_(1, 429)_ = 0.04, *p* = 0.833], so shortages of food products other than meat are not discussed.

**TABLE 1:** Associations between father’s education, meat shortage, and family type with morphometric and family traits of children and their parents.

Trait	Mean ± *SD* (*n*)	Mean ± *SD* (*n*)	Diff. betw. means	95% CI for diff. betw. means	*t* or *z*	*p*

	Father’s education: primary	Father’s education: above primary				
Residual height (*SD*)	−0.351 ± 1.069 (60)	0.041 ± 0.976 (541)	0.392	0.129, 0.655	2.93	0.004
Residual cranial vol. (*SD*)	−0.464 ± 0.929 (60)	0.050 ± 0.993 (537)	0.514	0.250, 0.778	3.83	0.0001
Mother’s age at birth (y)	27.7 ± 6.2 (52)	25.7 ± 5.1 (501)	3.0	0.5, 3.5	2.7	0.008
Mother’s height (cm)	164.3 ± 5.6 (55)	165.7 ± 5.3 (496)	1.4	−0.1, 2.9	1.8	0.070
Father’s height (cm)	176.6 ± 6.0 (46)	179.9 ± 6.7 (475)	3.3	1.3, 5.3	3.2	0.001
Birth weight (g)	3,375 ± 504 (47)	3,581 ± 541 (460)	206	44, 367	2.5	0.013
Number of siblings	1.45 ± 1.35 (60)	1.20 ± 0.96 (540)	0.25	−0.02, 0.52	0.7	0.501
Resource rating	3.08 ± 0.91 (59)	3.35 ± 0.76 (530)	0.27	0.06, 0.48	2.5	0.011

	**Meat shortage**	**No meat shortage**				

Residual height (*SD*)	−0.167 ± 1.061 (87)	0.003 ± 0.980 (585)	0.17	−0.05, 0.39	1.50	0.134
Residual cranial vol. (*SD*)	−0.308 ± 1.181 (87)	0.032 ± 0.972 (581)	0.34	0.11, 0.57	2.96	0.003
Mother’s age at birth (y)	27.5 ± 5.5 (83)	25.6 ± 5.2 (524)	1.9	0.7, 3.1	3.1	0.002
Mother’s height (cm)	165.2 ± 4.8 (83)	165.6 ± 5.5 (516)	0.4	−0.9, 1.7	0.6	0.551
Father’s height (cm)	179.8 ± 6.0 (73)	179.5 ± 6.7 (477)	0.3	−1.3, 1.9	0.4	0.726
Birth weight (g)	3,481 ± 618 (74)	3,566 ± 523 (475)	85	−46, 216	1.3	0.209
Number of siblings	1.43 ± 1.32 (87)	1.20 ± 1.02 (585)	0.23	−0.01, 0.47	1.1	0.271
Resource rating	2.61 ± 0.82 (87)	3.44 ± 0.73 (572)	0.83	0.66, 1.00	9.8	<0.0001

	**Birth-parents not together**	**Two birth-parents**				

Residual height (*SD*)	−0.060 ± 1.009 (249)	0.003 ± 0.985 (430)	0.063	−0.092, 0.218	0.80	0.422
Residual cranial vol. (*SD*)	−0.151 ± 0.995 (248)	0.060 ± 1.016 (427)	0.211	0.053, 0.369	2.61	0.009
Mother’s age at birth (y)	25.4 ± 5.2 (219)	26.1 ± 5.3 (393)	0.7	−0.2, 1.6	1.6	0.105
Mother’s height (cm)	165.9 ± 5.2 (210)	165.3 ± 5.5 (394)	0.6	−0.3, 1.5	1.4	0.164
Father’s height (cm)	180.0 ± 7.5 (166)	179.5 ± 6.3 (389)	0.5	−0.7, 1.7	0.8	0.399
Birth weight (g)	3,495 ± 533 (192)	3,584 ± 538 (360)	89	−5, 183	1.9	0.063
Number of siblings	1.03 ± 1.04 (247)	1.34 ± 1.06 (431)	0.31	0.15, 0.48	4.1	<0.0001
Resource rating	3.15 ± 0.77 (242)	3.44 ± 0.78 (419)	0.29	0.17, 0.41	4.7	<0.0001

	**Single provider**	**Two providers**				

Residual height (*SD*)	−0.017 ± 1.002 (170)	−0.021 ± 0.991 (509)	0.004	−0.169, 0.177	0.04	0.970
Residual cranial vol. (*SD*)	−0.095 ± 1.029 (169)	0.009 ± 1.007 (506)	0.104	−0.073, 0.281	1.16	0.248
Mother’s age at birth (y)	26.2 ± 5.4 (148)	25.8 ± 5.2 (464)	0.4	−0.6, 1.4	0.9	0.354
Mother’s height (cm)	166.5 ± 5.3 (144)	165.2 ± 5.4 (460)	1.3	0.3, 2.3	2.4	0.016
Father’s height (cm)	180.3 ± 7.2 (112)	179.4 ± 6.6 (443)	0.9	−0.5, 2.3	1.2	0.250
Birth weight (g)	3,539 ± 552 (132)	3,558 ± 533 (420)	19	−86, 124	0.4	0.722
Number of siblings	0.95 ± 1.08 (170)	1.32 ± 1.04 (508)	0.37	0.19, 0.55	4.6	<0.0001
Resource rating	3.03 ± 0.76 (165)	3.43 ± 0.77 (496)	0.4	0.3, 0.5	5.7	<0.0001

	**Birth-parent + step-parent**	**Two birth-parents**				

Residual height (*SD*)	−0.161 ± 0.980 (104)	0.003 ± 0.985 (430)	0.164	−0.047, 0.375	1.53	0.128
Residual cranial vol. (*SD*)	−0.212 ± 0.924 (104)	0.060 ± 1.016 (427)	0.272	0.057, 0.487	2.49	0.013
Mother’s age at birth (y)	23.6 ± 4.5 (91)	26.1 ± 5.3 (393)	2.5	1.3, 3.7	4.23	<0.0001
Mother’s height (cm)	165.3 ± 5.3 (85)	165.3 ± 5.5 (394)	0	−1.3, 1.3	0.01	0.962
Father’s height (cm)	179.6 ± 8.0 (68)	179.5 ± 6.3 (389)	0.1	−1.6, 1.8	0.20	0.250
Birth weight (g)	3,419 ± 512 (77)	3,584 ± 538 (360)	165	33, 296	2.47	0.014
Number of siblings	1.00 ± 0.97 (102)	1.34 ± 1.06 (431)	0.34	0.11, 0.57	3.31	0.003
Resource rating	3.34 ± 0.71 (101)	3.44 ± 0.78 (419)	0.1	−0.1, 0.3	1.13	0.258

**All p-values for differences between groups except for the number of siblings (that is tested via U-test) are from t-tests. Residual height and cranial volume are in standard deviation units, i.e., age- and sex-specific values transformed to z-scores within sexes. Meat shortage (yes/no) and resource rating (6-point scale, 0–5) are based on the self-reports of children. Birth-parents not together vs. Two birth-parents compares single parent + stepfamilies against families where both biological parents are present. Single provider vs. Two providers compares children living with a single parent (predominantly mother) against children living either with two birth-parents or with one birth-parent and a step-parent. Birth-parent + step-parent vs. Two birth-parents compares children in step-families (with two providers) against children living with two birth-parents.**

**TABLE 2:** Associations between age-adjusted residual height and cranial volume with morphometric and family traits of children and their parents in ANCOVA.

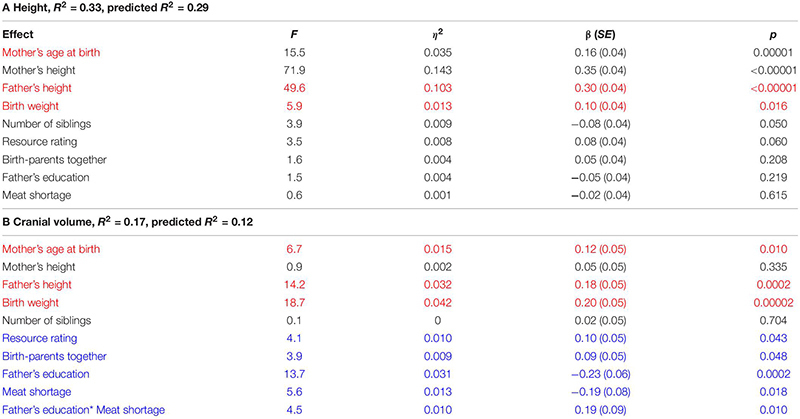

**Residual height and cranial volume are in standard deviation units, i.e., age-specific values transformed to z-scores within sexes. Last three predictors are factors with two levels; other predictors are continuous. “Birth-parents together” (coded as 1) compares children living with both birth-parents against all other family types (single-parent and step-families coded as 0). “Father’s education” is coded as 1 if the father had primary education and 0 if the father had secondary or tertiary education. “Meat shortage” is coded as 1 if the children reported meat shortage and 0 if they did not report it. N = 440 for height and 436 for cranial volume. Resource rating is given at 6-point scale (0–5). Units for continuous predictors are shown in the first column of Table 1. η^2^ is a partial η^2^, a measure of effect size (variance explained by a given variable of the variance remaining after excluding variance explained by other predictors). β is a standardized regression coefficient. Predictors that had a similar effect on height and cranial volume are indicated in red; predictors that affected only cranial volume are indicated in blue.**

### Assessment of Resource Availability

The second specific question concerned resource availability: “How do you rate the economic situation in the family?” (5—very good, 4—good, 3—satisfactory, 2—poor, 1—very poor). This variable was termed as resource rating; its distribution is depicted in Supplementary Figure S1. in the ESM. Resource rating likely captured some important aspect of resource availability as average resource rating was always significantly lower in families where the children had reported either shortage of meat (Table 1) or milk, fruits, or sweets (Supplementary Table S1). On the other hand, since resource rating was measured as subjective self-rating, it may be highly unreliable as different subjects may have different standards for how they rate resource availability, especially because the subjects were teenagers. We therefore examined correlations between resource rating and two other semi-quantitative measures of resource availability, namely the parent-reported monthly family income and family income divided by the number of family members (Supplementary Table S2). The correlations were not particularly strong (*r*_*s*_ = 0.45 for resource rating vs. family income and *r*_*s*_ = 0.27 for resource rating vs. family income per person). At that, reporting of family income was highly biased: only 5.6% (14/249) of single parents reported their income while virtually all (430/431) families with two providers did so. This (as well as the highly ordinal nature of this variable) precluded the use of income in the models testing the associations between anthropometric traits and family structure.

### Statistical Analyses

Binary associations between metric traits were analyzed by Pearson correlation, *t*-tests, *U*-tests, and *χ*^2^-tests. ANCOVAs were used for testing the simultaneous effects of mediating and confounding variables on growth. In these models, either height or cranial volume of a child was a dependent variable and heights of mother and father, mothers’ age at birth, number of siblings, and resource rating were entered as continuous predictors. Family structure, father’s education, and meat shortage were entered as binary factors; the coding is explained in the headings of Tables 1, 2. Models in Table 2 did not reveal multicollinearity (VIFs ranged from 1.1 to 3.9; according to Kutner et al. (2005), VIF values < 10 are typically considered acceptable for most purposes) or overfitting (see *R*^2^ vs. predicted *R*^2^ values in the table). Adding school IDs did not predict variation in morphometric traits in the models in Table 2 [height: *F*_(5, 425)_ = 1.4, *p* = 0.219; cranial volume: *F*_(5, 425)_ = 2.0, *p* = 0.075]; hence this variable was omitted from the models. Parental cranial volumes were not used as a control variable due to the lack of these data. Due to the exploratory nature of the study, we did not rely on significance thresholds for interpreting test results. However, we report *P*-values along with test statistics and measures of effect size for the ease of assessment of the evidence against the statistical null hypothesis (Amrhein et al., 2017). It should be noted, however, that in exploratory research, *P*-values have unknown diagnosticity and that their use can falsely imply testing rather than generating hypotheses (Nosek et al., 2018). For assessing effect sizes, we present eta-squared values for each predictor in Table 2 and provide 95% CI for the difference between means for bivariate comparisons in Table 1. Sample sizes vary between analyses due to incomplete response rate among the participants.

## Results

### Bivariate Associations Between Height, Cranial Volume, and Characteristics of Growth Environment

Inspection of bivariate associations between metric traits (Supplementary Figure S1) revealed that residuals of height and cranial volume were moderately intercorrelated (*r* = 0.46) and that both measures of size correlated positively with parental heights and birth weight. Height but not cranial volume correlated positively with maternal age at birth and negatively with the number of siblings. In the whole sample, height and cranial volume had similar associations with income per family member (height: *r*_*s*_ = 0.17, *n* = 468, *p* = 0.0002; cranial volume: *r*_*s*_ = 0.13, *n* = 464, *p* = 0.005). The correlation between income and height was roughly similar among boys and girls (boys, height: *r*_*s*_ = 0.19, *n* = 218, *p* = 0.005; girls, height: *r*_*s*_ = 0.14, *n* = 250, *p* = 0.028). However, the correlation between income and cranial volume was stronger among boys (*r*_*s*_ = 0.18, *n* = 218, *p* = 0.008) than among girls (*r*_*s*_ = 0.08, *n* = 246, *p* = 0.202). Differently from height, cranial volume correlated positively with self-rated resource availability (Supplementary Figure S1). This association was similar among both boys and girls (*r* = 0.12). Parents mated assortatively with respect to height (*r* = 0.26), children with more siblings were slightly heavier at birth (*r* = 0.10), and taller mothers had fewer children (*r* = 0.09). Children born to older mothers tended to be less satisfied with resource availability (*r* = −0.08).

**FIGURE 1:**
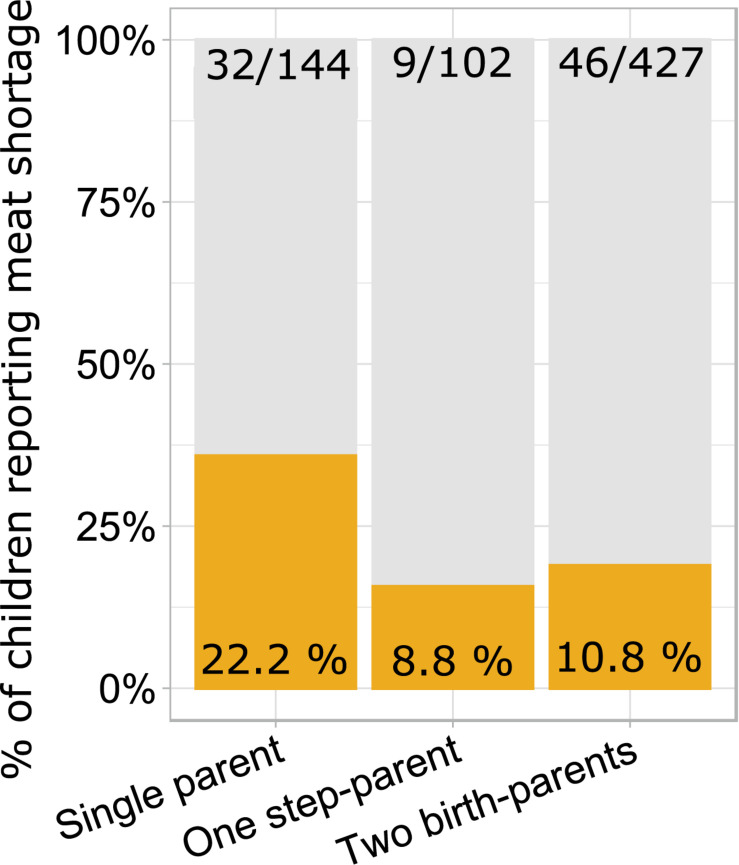
Proportions and numbers of children reporting meat shortage in relation to family type. Single-parent families have a higher proportion of children reporting meat shortage than two-provider families with a step-parent (*χ*^2^ = 7.7, *p* = 0.005) or two birth-parents (*χ*^2^ = 14.3, *p* = 0.0008). The latter two categories do not differ from each other (*χ*^2^ = 0.3, *p* = 0.562).

Some but not all risk factors of poor growth were aggregated. Children of single mothers reported meat shortage more often than children living with birth-parents or one step-parent while the presence of a step-parent was not associated with reported meat shortage in families with two providers (Figure 1). On the other hand, meat shortage was not more prevalent among children of fathers with primary education (18%, 11/60) than among children of fathers with secondary and tertiary education (13%, 68/540; *χ*^2^_1_ = 1.6, *p* = 0.212). Fathers with primary education were more prevalent (13%; 27/201) in families including a step-parent than among children who lived with both birth-parents (8%, 33/401; *χ*^2^_1_ = 4.0, *p* = 0.044). Prevalence of fathers with primary education did not differ between single-parent families (12.5%, 16/128) and families with two providers (9%, 44/474; *χ*^2^_1_ = 1.2, *p* = 0.281).

**FIGURE 2:**
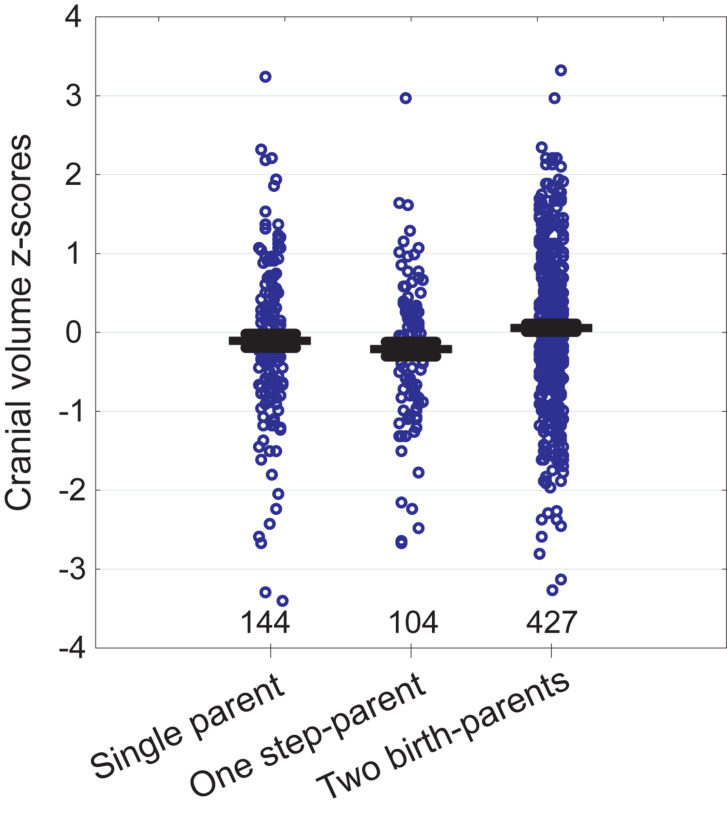
Cranial volume of children in relation to family type. Whiskers denote standard errors. Sample sizes at bottom.

In bivariate tests (Table 1), children of fathers with primary education were shorter, had smaller heads, weighed about 200 g less at birth, and reported lower resource availability than children of fathers with secondary or tertiary education. Fathers with primary education were themselves on average 3.3 cm shorter than more highly educated fathers. Children who reported meat shortage had smaller heads but were not shorter than those who did not report meat shortage. Children living with both birth-parents had larger heads than those living in families including a step-parent (see also Figure 2). Height was not associated with the number of providers and children of single parents did not have smaller heads than children from the families with two providers. Children living with two birth-parents or two providers had more siblings and they were more satisfied with their resource availability than children from single-parent or step-parent families.

### Height vs. Growth Environment, Controlling for Covariates

In an ANCOVA accounting for all variables that were associated with growth in bivariate models, only parental heights, mother’s age at birth, and birth weight were associated with children’s height. Accounting for these confounders, number of siblings showed a weak negative association with height while the resource rating had a positive association with height of the same magnitude. Family structure, paternal education, and reported meat shortage did not reveal any sizeable associations with height of children when boys and girls were analyzed together. When boys and girls were analyzed separately, no substantial associations between height and variables related to growth environment could be detected (Supplementary Table S3).

### Cranial Volume vs. Growth Environment, Controlling for Covariates

**FIGURE 3:**
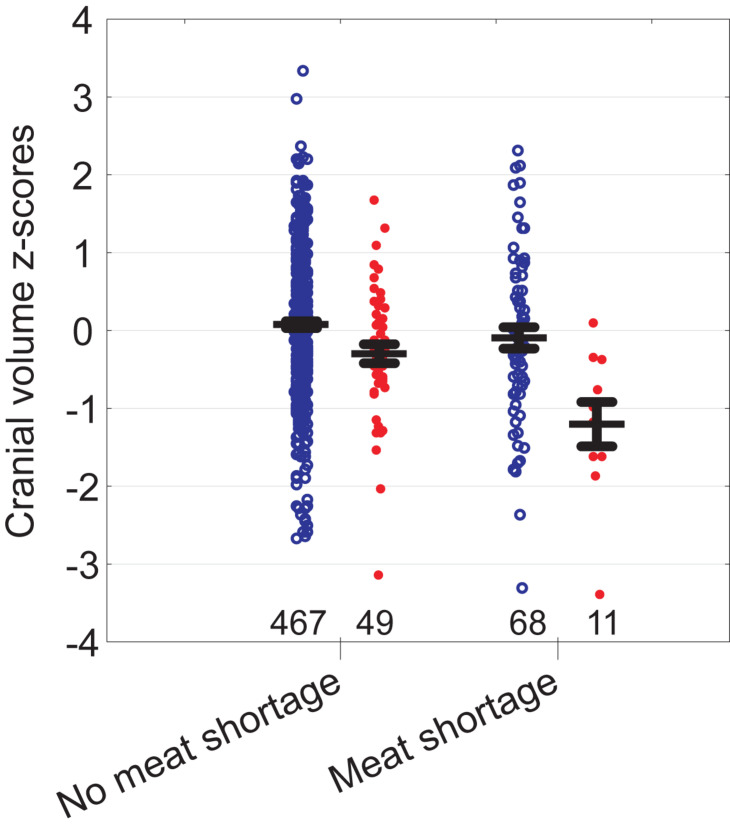
Cranial volume of children in relation to self-reported meat shortage and paternal education (red filled symbols—primary education, blue open symbols—education above primary). Whiskers denote standard errors. Sample sizes at bottom.

Cranial volume was associated with all predictors in the model except mother’s height and the number of siblings (Table 2B). The strongest associations were detected between cranial volume and birth weight, father’s height, and education. We also detected an interaction for father’s education and meat shortage: self-reported meat shortage had a particularly strong association with the cranial volume of children in families where the father had only primary education (see Figure 3). The association of family type with cranial volume was specific to whether the children lived with both birth-parents but not whether they had two providers: when the term “Two birth-parents” in the model in Table 2B was substituted with “Two providers,” no significant association with being reared by a single parent was found [*F*_(1, 425)_ = 0.7, *p* = 0.397]. In bivariate tests, children living with both birth-parents had larger crania (0.27 *SD* units, 95% CI = 0.06–0.49) than those living with a step-parent, while other groups did not differ from each other with respect to cranial volumes (Figure 2). It should be noted, however, that living with two birth-parents explained only 1% of the variance in cranial volume in the model (Table 2B). When the same model was tested separately for boys, no associations between cranial volume and characteristics of growth environment could be detected. In the case of girls, the associations between cranial volume, paternal education, and meat shortage were similar as in the analyses involving all children (Supplementary Table S3). It should be noted, however, that the sample size for boys who reported meat shortage and had fathers with primary education was only four (Supplementary Figure S2). The current dataset is thus insufficient for detecting sex differences in interactive effects of paternal education and meat shortage on cranial volume.

## Discussion

The results of this study demonstrate how constraints associated with parental investment relate to the body and head growth of children in a European country in the transition from the Soviet regime to a market economy at the end of the twentieth century. Three of these constraints had similar associations with both head and body growth: children who were born to younger mothers, weighed less at birth, and had shorter fathers were relatively shorter and had smaller heads than their peers of the same age (Table 2). This finding is consistent with previous studies demonstrating positive associations between maternal age, birthweight, and offspring stature (reviewed in Galobardes et al., 2012); however, to our knowledge, the associations of these factors with adolescent cranial volume have not been reported previously. Strong associations between parents’ and children’s height obviously reflect a genetic contribution, as height has heritability estimates around 0.8 (Mayhew and Meyre, 2017). Associations between birth weight and body and head dimensions likely reflect shared genetic architecture between height and head size (see Posthuma et al., 2000) and also long-lasting effects of maternal somatic investments through fetal growth (Haeffner et al., 2002; Addo et al., 2015) which, in turn, can markedly affect later development (Gale et al., 2004). It should be recalled, however, that growth environment itself has a substantial genetic component (Vinkhuyzen et al., 2010).

### Height

Body height was associated with a smaller number of growth constraints than cranial volume, the main exception being the number of siblings, which had a weak association only with height in bivariate analysis (*r* = −0.13 vs. *r* = −0.02 in the case of cranial volume). The negative association between the number of siblings and height reflects the central life-history trade-off between offspring number and quality. Similar patterns have been repeatedly observed in modern wealthy, well-nourished populations of low fertility and mortality (reviewed in Li et al., 2004; Lawson and Mace, 2008; Krams et al., 2019).

The nature of this trade-off cannot be explained on the basis of current data. In the case of a microevolutionary trade-off, the association between offspring height and number would emerge because genetically tall people are genetically predisposed to have smaller families (as shown for women but not men in United Kingdom (Sanjak et al., 2018) and United States (Beauchamp, 2016; Conley et al., 2016). In the case of a physiological trade-off, access to the resources required for normal growth would constrain the height of children in large families, provided that these resources are limiting and thus become diluted between siblings. The positive correlation between height and income per family member (*r*_*s*_ = 0.17) is consistent with such a resource dilution model. However, it is also noteworthy that the correlation between the number of siblings and children’s self-reported resource rating was weak (*r* = −0.07) and that height was not associated with meat shortage, living with birth-parents, or the number of providers (Tables 1, 2).

### Cranial Volume

In the bivariate analysis, both mother’s and father’s height predicted the cranial volume of their children (*r* = 0.15 and 0.23, respectively; Supplementary Figure S1). However, in the ANCOVA model, only father’s height retained a sizeable effect (Table 2B). This finding is notable as it suggests that the genetic component of the linear size of parents is not uniformly reflected in the head size of their children—otherwise we should have detected a simultaneous effect of both parents’ height on cranial volume. We are not aware of any previous documentation of such specifically paternal effects on cranial growth of adolescents, although positive correlations of maternal and paternal height with newborn skeletal measurements like length and head circumference are well described (Leary et al., 2006; Wills et al., 2010).

Fathers with primary education were on average 3.3 cm shorter than fathers with an education over primary. Yet the association between father’s education and cranial volume of their offspring remained detectable in a model accounting for all measured mediators and confounders, and its magnitude was as strong as that of father’s height (Table 2B). It thus appears that fathers’ education captures a substantial proportion of the association between parental quality and cranial growth of their offspring. This finding is consistent with anthropometric (Gale et al., 2004) and brain imaging studies demonstrating lasting associations between parental socioeconomic status and/or education vs. brain structure, growth, development, and functioning (reviewed by Leijser et al., 2018; LeWinn and Shih, 2019). The current study does not enable distinguishing whether the association between paternal education and children’s cranial volume was primarily of environmental or genetic origin. Although improvement of the growth environment can enhance head growth (Hõrak and Valge, 2015; Leijser et al., 2018; LeWinn and Shih, 2019), it is also well established that most variables traditionally thought of as markers of the quality of growth environment also reflect genetic variability (e.g., Turkheimer et al., 2003). For instance, the genetic correlation between household income and infant intracranial volume in the UK Biobank sample was 0.53 (Hill et al., 2016). The heritability of head size in adolescence is also remarkably high (0.83–0.87; Smit et al., 2010).

Unlike height, cranial volume was associated with self-reported meat shortage in both bivariate analysis and ANCOVA adjusting for mediators and confounders. Previously, effects of undernutrition on head growth in infancy have been well established in developing societies (reviewed in Ivanovic, 1996; Martorell and Zongrone, 2012; Miller et al., 2016) and in very low birth weight preterm infants in developed countries (Hayakawa et al., 2003; Morgan et al., 2014; Belfort et al., 2019). A Swedish study showed that infants of mothers with a history of eating disorders had delayed head growth until at least 18 months of age while height of infants was not associated with maternal condition (Koubaa et al., 2013). Cranial volume predicts brain size, intelligence, and educational attainment (reviewed by Valge et al., 2019; Jansen et al., 2020) and positive effects of nutritional supplementation on intelligence or achievement have been reported in lactants, school children, and adolescents as well as pregnant mothers in both developed or developing countries (reviewed by Arija et al., 2006; DiGirolamo et al., 2020). Further, even in developed countries, the surplus of dietary energy is not always matched with proportional availability of micronutrients, and some micronutrients, including iron, are particularly sensitive to marginal dietary intake, since their needs increase substantially during adolescent growth (Caballero, 2002; Arija et al., 2006). Importantly, meat is the best source of easily absorbable heme iron (Abbaspour et al., 2014) and also an excellent source of zinc (Kristensen et al., 2006). Both minerals play a crucial role in head growth (Krebs et al., 2006; Surkan et al., 2012), brain development (Wang et al., 2019), and functioning (Stoecker et al., 2009; Johnson et al., 2016). For instance, among Kenyan children (with average age of 7.4 years), meat supplementation had significant effects on cognitive, social, and physical abilities while it did not affect linear growth (Neumann et al., 2007).

It is thus possible that the findings of the current study reflect the direct positive effect of meat consumption on brain development for which the age-specific cranial volume appears as a sensitive indicator. On the other hand, caution is required with such an interpretation because the actual meat consumption or its biomarkers were not measured. We thus cannot exclude the possibility that self-reported meat shortage appears primarily as a proxy for some unmeasured marker of general quality of parental investment in a broader sense.

In this context it is noteworthy that in addition to the overall (main) effect of meat shortage on cranial volume, we also detected its interaction with paternal education (Table 2B). That is, compared to children whose father had at least secondary education, children whose father had only primary education were particularly sensitive to meat shortage (Figure 3 and Supplementary Figure S2). Particular developmental vulnerability of children with low paternal education has been also demonstrated elsewhere. In a Taiwanese study, lower paternal education level tended to worsen the negative impact of low birth weight on cognitive test scores (Wang et al., 2008). In England in the 1970s, sibling number was negatively associated with the height of children whose fathers were manual workers while the height of non-manual workers’ children was not associated with family size (Rona et al., 1978). We thus cannot exclude the possibility that children of more educated fathers were buffered from the effect of meat shortage on head growth by some beneficial effects characteristic to such families (e.g., social stimulation; see below). For instance, in a United Kingdom birth cohort of 1991–92, involvement of fathers in parental care increased with family socioeconomic position (Lawson and Mace, 2009). Another possible explanation would be that children of less educated fathers were less prone to complain and accordingly, reported meat shortage only in severe cases, while children of more educated fathers tended to report also less acute shortages. Finally, it may be noted that the interaction between parental education and meat availability is consistent with the Scarr-Rowe effect (the interaction between the heritability of IQ and parental SES; see Figlio et al., 2017). This is because among offspring from lower SES households with poorly educated fathers, the heritability of cranial volume might be expected to be suppressed by environmental factors, which might account for the direction of the interaction with meat availability.

### Importance of Family Structure

Children living with both birth-parents had larger crania than those living in families where one of the providers was a step-parent. This association was detectable in both bivariate analysis (Table 1 and Figure 2) and in an ANCOVA adjusting for mediators and confounders (Table 2B), although, notably, the effect size was very small. At the same time, cranial volumes of children living with a single parent were similar to those living with two providers, even though the former reported on average lower resource availability (Table 1) and more frequent meat shortage (Figure 1). Associations between family type and cranial volume thus cannot be explained on the basis of dilution of material resources. However, the observed difference in cranial volumes between children living with step-parents vs. birth-parents is compatible with the evolutionary view of parenting, stating that investment into step-children is against the genetic interest of parents because it would divert resources away from their own progeny (Daly and Wilson, 1980; Mace, 2015). Resources in this context comprise more than just material wealth and include any type of parenting activities and time spent interacting with children. Lawson and Mace (2009) showed in a sample of British children (born 1991–92) that parenting activities of single mothers were higher than those of mothers who lived with the biological father of their children and that children living with a nonbiological father received the least amount of maternal time investment. Hence, mothers reduced investment in offspring from former partners only if a new partner was present. By the age of 10, children of single mothers were shorter than those who lived with their genetic parents but they were still taller than children from families with a new father figure (Mace, 2015).

In the current study, we did not detect any associations between family type and the height of children (which may result from the limited test power as our sample size was 18 times smaller than that of the British study by Lawson and Mace, 2009). However, we detected a negative association between living with a step-parent and cranial growth. The proximate explanation for such an association involves psychosocial stress resulting from low parenting effort. For instance, a study in a normal Dutch population showed that higher levels of sensitive parental care (characterized by prompt and adequate response to the child’s signals and needs) in early childhood were associated with larger total brain volume at 8 years, controlling for infant head size (Kok et al., 2015). A possible mechanism here involves sensitivity of growth hormone/IGF-1 pathway to psychological stress (Gohlke et al., 2004; Johnson et al., 2010).

An alternative (yet not mutually exclusive) explanation to the observed associations between family type and cranial volume of children would be that parents prone to remarrying possess on average (genetically) smaller heads than those prone to avoiding divorce or remaining single after divorcing. Such a scenario would assume robust genetic correlations between cranial volume and personality traits related to marriage stability. Twin studies have shown that genetic factors account for 13–53% of the variation in divorce (reviewed in Salvatore et al., 2018), and if personality traits associated with a propensity to divorce are genetically correlated with cranial volume or its growth rate, one would detect smaller heads of children growing up in divorced/separated families. Such an explanation would be consistent with the predictions of life history theory, assuming that qualities characteristic of slow pace of life—including high somatic investment into body and brain growth and propensity for relatively low mating effort (in relation to parenting effort)—have coevolved (and cluster) with higher mental abilities and conscientious and risk-averse personality traits (Rushton, 1985; Figueredo et al., 2006, 2014; Luoto, 2019a). Consistent with this view are also the findings in our sample where fathers with only primary education were shorter and more prone to divorce/separate than others.

It should be noted, however, that the original contention of Rushton (1985), that general intelligence (*g*) should constitute a component of fast-slow life history continuum because *g* is related to brain size, and bigger brains are more expensive in terms of somatic effort (hence should have been favored under conditions promoting slower as opposed to faster life histories), has been questioned by studies assessing phenotypic and genetic correlations between *g* and psychometrically assessed life-history speed (Woodley, 2011; Woodley et al., 2013; Woodley of Menie and Madison, 2015). On the other hand, one should perhaps not ignore the possibility that dysgenic selection via educational attainment may lead to coupling of (at least some) traits considered characteristic to fast life histories (such as smaller heads and bodies) with high reproductive rates. For instance, taller Estonian girls with larger heads were more likely to proceed to secondary and/or tertiary education during the past century (Valge et al., 2019), ending up with delayed reproduction and lower lifetime reproductive success (Valge et al., 2020).

### Sex Differences

Correlations between height and income per family member were roughly similar among boys and girls (boys: *r*_*s*_ = 0.19; girls: *r*_*s*_ = 0.14). This result differs from findings of two studies performed in Latvia in 2010 where income per family member correlated positively with height in boys (Krams et al., 2019) but not in girls (Rubika et al., 2020). However, we found that cranial volume was more strongly associated with income among boys (*r*_*s*_ = 0.18) than among girls (*r*_*s*_ = 0.08). The latter finding is consistent with the idea that males are less buffered against the environmental effects on growth and development (Stinson, 1985) and suggests that cranial volume may be a more sex-specific marker of resource availability than height.

## Conclusion, Limitations, and Implications

A noteworthy finding of this study is that cranial growth is particularly sensitive to the quality of growth environment, as it was associated with higher number of growth constraints than height. We showed for the first time that self-reported meat shortage and living with a step-parent are associated with smaller cranial dimensions in a sample of 11–17-year-old children in a European country undergoing an economic transition. In addition, children of fathers with only primary education had smaller heads, particularly when such children self-reported meat shortage. However, family type, paternal education, and self-reported meat shortage were not associated with the stature of children in the same dataset, indicating that cranial volume is a more sensitive marker of the selected growth constraints/parental investment than height. At first, such a pattern may be difficult to reconcile with the concept of thrifty phenotype (Hales and Barker, 1992; Wells, 2013) and predictions of the hypotheses stating that under resource limitation, brain growth is prioritized over that of the body (Kuzawa et al., 2014; Said-Mohamed et al., 2018). It should be recalled, however, that these hypotheses do not necessarily predict that buffering brain growth at the expense of body growth extends beyond the period of fast brain development during infancy/early childhood. It should be also considered that in the current study, size is a marker of the magnitude of growth that has not yet been completed. That is, both height and head are still increasing through late childhood and adolescence, and might be doing so at different rates. The variability in phenotype present at this age may not necessarily remain at adulthood, and it is possible that linear growth and cranial traits show differential sensitivity to external constraints in different periods of childhood and/or adolescence.

Our results are consistent with findings which have shown that even in calorie-sufficient populations, micronutrient availability may constrain brain growth and development (e.g., Arija et al., 2006; Caballero, 2002), affect cognitive ability (Lassek and Gaulin, 2008, 2015), and that higher levels of sensitive parental care in childhood are associated with larger brain volumes (Kok et al., 2015). These findings have practical implications for public health policy as childhood cranial volume is an important predictor of future cognitive abilities and educational attainment (reviewed in Valge et al., 2019).

One of the serious limitations of the current study is an absence of objective measures of material resource availability for the studied children. Their self-reported resource availability rating correlated only modestly with semi-quantitative estimates of total family income and income per person (Supplementary Table S2). Reporting of family income was, however, highly biased as only 6% of single parents reported their income. This (as well as the highly ordinal nature of this variable) precluded the use of income in the models testing the associations between anthropometric traits and family structure. Another important limitation of this study is inherent to all non-genetic studies of human growth, i.e., our inability to distinguish whether the established effects of parental investment on growth reflect primarily external constraints or microevolutionary trade-offs. For instance: are experiencing meat shortage or having a poorly educated father traits that are genetically independent of the trait of having a small head? Such a scenario would mean that growth is constrained only by availability of external resources. Or, is there a reason to expect genetic clustering of traits associated with fast pace of life (e.g., smaller heads, lower education/income and propensity for behavior that is not compatible with sustained parental investment)? Such issues would have been easier to address if we had measures of parental cranial volumes at our disposal. We hope that the questions about genetic clustering of life-history, behavioral, and anthropometric traits can be addressed more efficiently by investigating more comprehensive anthropometric datasets and in studies of genome-wide associations (Day et al., 2016) and genetic polymorphisms (Minkov and Bond, 2015; Luoto, 2019b) that enable measuring genetic correlations between the sets of variables associated with pace of life.

The finding that head size appears more sensitive to family structure (which is likely associated with cognitive and emotional stimulation) than height might seem intuitive. However, we are not aware of any previous reports of such associations, so the generality of our results awaits further confirmation in other populations. Nonetheless, our study highlights the importance of measuring cranial volume as a sensitive marker of growth environment and suggests that such an approach would enable quantifying the physical impact of non-material parental investment, a topic largely neglected in human behavioral ecology.

## Data Availability Statement

The original contributions presented in the study are included in the article/Supplementary Material, further inquiries can be directed to the corresponding author/s.

## Ethics Statement

The studies involving human participants were reviewed and approved by the Research Ethics Committee of the University of Tartu. Written informed consent from the participants’ legal guardian/next of kin was not required to participate in this study in accordance with the national legislation and the institutional requirements.

## Author Contributions

GV designed and performed the original study. VL contributed to data digitalisation. PH and VL designed the prospective study, analyzed the data and wrote the manuscript. All authors contributed to the article and approved the submitted version.

## Conflict of Interest

The authors declare that the research was conducted in the absence of any commercial or financial relationships that could be construed as a potential conflict of interest.
