# Supplementary material for: Adolescent Cranial Volume as a Sensitive Marker of Parental Investment: The Role of Non-material Resources?
Source: Front Psychol. 2020 Dec 15;11:602401. doi: 10.3389/fpsyg.2020.602401 (PMC7769954; doi:10.3389/fpsyg.2020.602401)
Supplement: Supplementary file 1 [file Data_Sheet_1.PDF]

Electronic Supplementary Material ESM

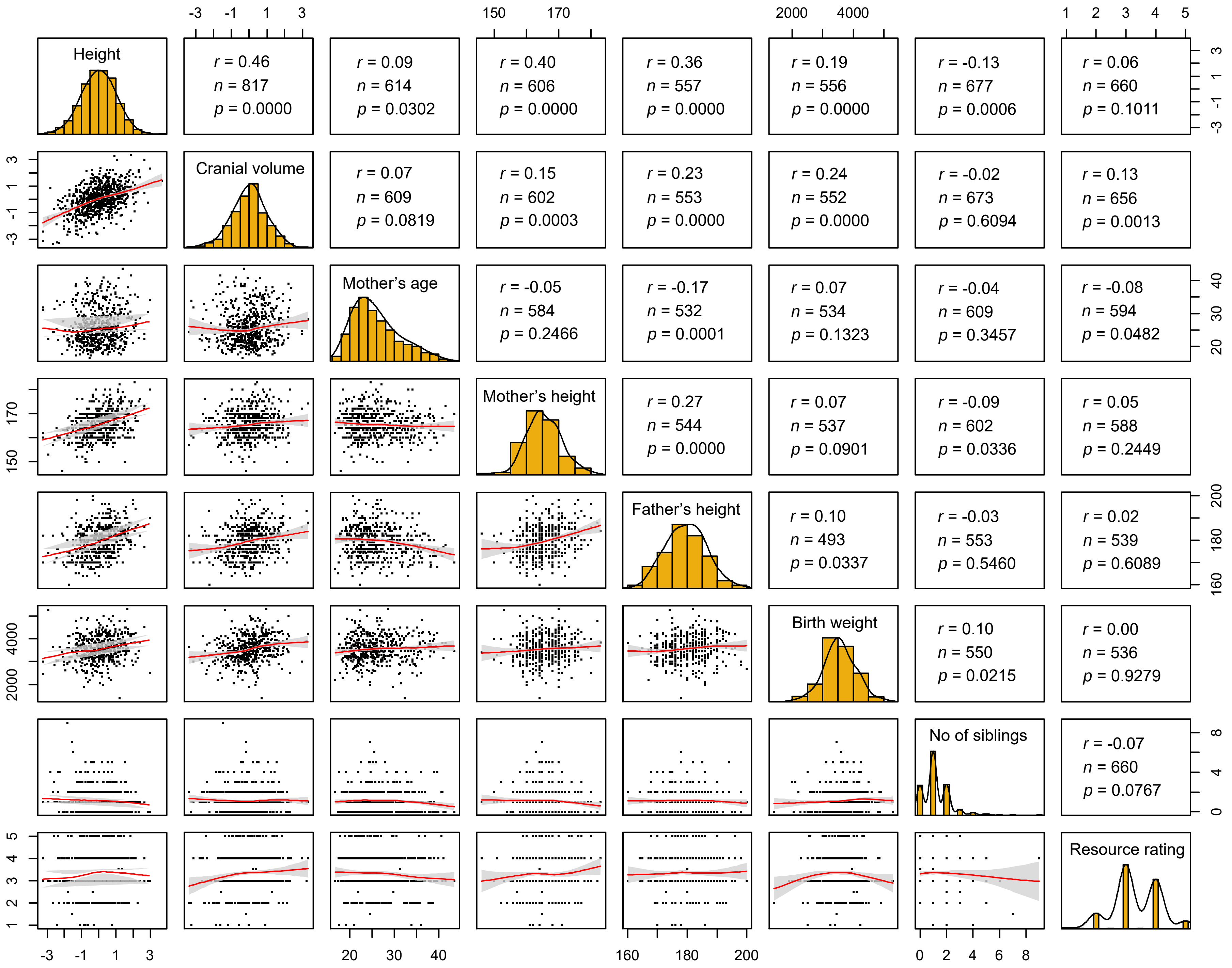

Fig. S1. Trait distributions (on diagonal), Pearson correlations between the metric variables used in the analyses (above diagonal) and scatterplots with lowess-smoothed regression lines with 95% CI (below diagonal).

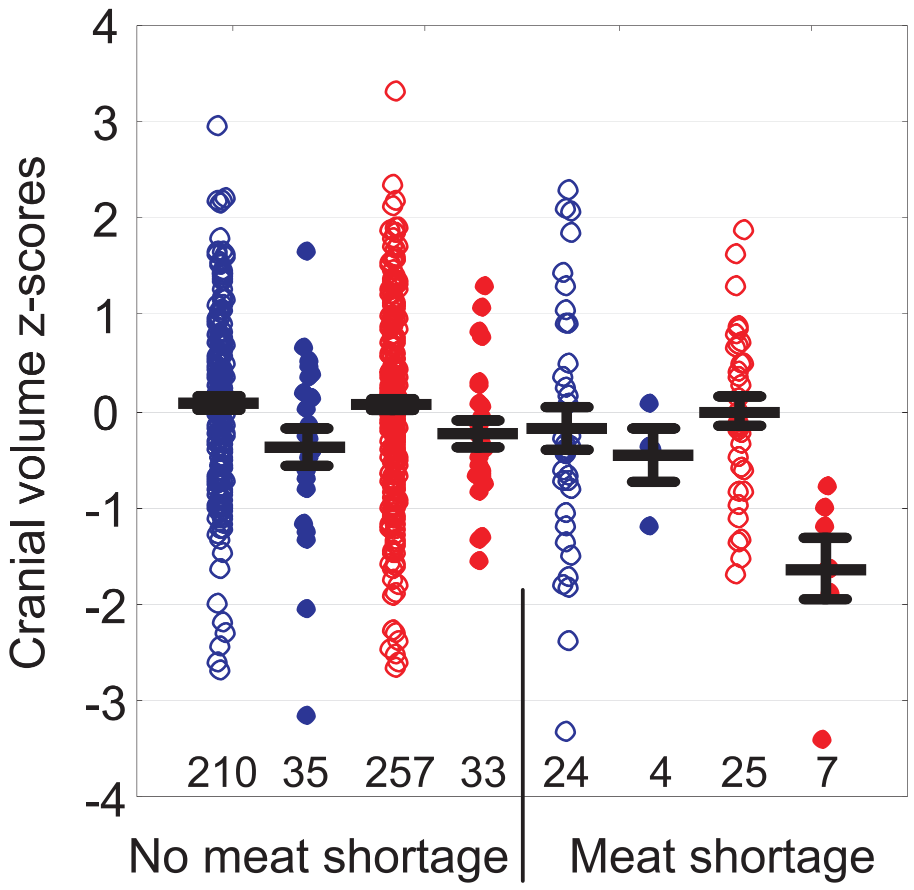

Fig. S2. Cranial volume of children in relation to family type. Whiskers denote standard errors. Sample sizes at bottom. Blue – boys, red – girls. Open symbols – fathers with secondary or tertiary education, filled symbols – fathers with primary education.

Table S1. Associations between meat, fruit and sweet shortage with morphometric and family traits of children and their parents. All pairwise differences except the number of siblings (that is tested via *U*-test) are from *t*-tests. Residual height and cranial volume are in standard deviation units, i.e. age- and sex-specific values transformed to *z*-scores within sexes. Food item shortages (yes/no) and resource rating (0 to 5) are based on the self-reports of children. *P*-values below 0.05 are in bold.

Trait	Mean ± *SD* (*n*)	Mean ± *SD* (*n*)	*t* or *z*	*p*
	Milk shortage	No milk shortage		
**Residual height (*SD*)**	**-0.284 ± 0.986 (65)**	**0.010 ± 0.989 (607)**	**2.3**	**0.023**
Residual cranial volume (*SD*)	-0.195 ± 0.984 (60)	0.007 ± 0.984 (603)	1.5	0.125
**Mother's age at birth (years)**	**27.4 ± 5.5 (61)**	**25.7 ± 5.2 (546)**	**2.4**	**0.018**
Mother's height (cm)	164.4 ± 4.6 (60)	165.6 ± 5.5 (496)	1.6	0.104
Father's height (cm)	178.1 ± 6.3 (54)	179.7 ± 6.6 (496)	1.7	0.090
Birth weight (g)	3514 ± 597 (50)	3558 ± 531 (499)	0.5	0.583
Number of siblings	1.39 ± 1.15 (65)	1.21 ± 1.04 (607)	1.1	0.226
**Resource rating**	**2.74 ± 0.85 (65)**	**3.40 ± 0.76 (594)**	**6.6**	**<0.0001**
	Fruit shortage	No fruit shortage		
Residual height (*SD*)	-0.038 ± 0.927 (121)	-0.031 ± 1.006 (585)	0.7	0.492
Residual cranial volume (*SD*)	0.050 ± 1.022 (120)	-0.026 ± 1.004 (548)	0.7	0.453
**Mother's age at birth (years)**	**26.9 ± 5.6 (115)**	**25.7 ± 5.1 (492)**	**2.4**	**0.014**
Mother's height (cm)	165.3 ± 5.8 (113)	165.6 ± 5.3 (516)	0.5	0.608
Father's height (cm)	179.5 ± 6.3 (102)	179.6 ± 6.7 (448)	0.1	0.917
Birth weight (g)	3541± 549 (103)	3557 ± 534 (446)	0.3	0.786
**Number of siblings**	**1.43 ± 1.23 (122)**	**1.18 ± 1.01 (550)**	**1.9**	**0.047**
**Resource rating**	**2.97 ± 0.71 (122)**	**3.41 ± 0.79 (537)**	**3.4**	**<0.0001**
	Sweets shortage	No sweets shortage		
Residual height (*SD*)	-0.040 ± 1.015 (61)	-0.016 ± 0.990 (611)	0.2	0.858
Residual cranial volume (*SD*)	-0.098 ± 1.195 (61)	-0.004 ± 0.987 (607)	0.7	0.484
Mother's age at birth (years)	27.1 ± 5.9 (54)	25.8 ± 5.2 (553)	1.7	0.085
Mother's height (cm)	165.4 ± 6.1 (53)	165.5 ± 5.3 (394)	0.1	0.894
Father's height (cm)	180.5 ± 7.4 (46)	179.5 ± 6.6 (389)	1.0	0.316
Birth weight (g)	3503 ± 538 (47)	3559 ± 537 (502)	0.7	0.500
Number of siblings	1.28 ± 0.97 (61)	1.22 ± 1.07 (611)	0.5	0.580
**Resource rating**	**2.90 ± 0.80 (60)**	**3.38 ± 0.77 (599)**	**4.5**	**<0.0001**

Table S2. Spearman rank correlations (r_s (n) p) between self-reported resource availability and estimates of income. (Monthly) family income was recorded on ordinal scale (transformed from Estonian Kroon to EUR) as <32, 32-64, 64-128, 128-192, 192-256, 256-320, 320-639 and >639. Income per person was calculated as average of the income interval divided by the number of family members. Resource rating was derived from the response to the question "How do you rate the economic situation in the family?" (5 – very good, 4 – good, 3 – satisfactory, 2 – poor, 1 – very poor).

Boys + girls	
Family income	Income per person
0.45 (464) <0.00001	0.27 (463) <0.00001
Boys	
0.35 (216) <0.00001	0.14 (216) 0.034
Girls	
0.52 (248) <0.00001	0.36 (247) <0.00001

Table S3. Sex-specific associations between age-adjusted residual height and cranial volume with morphometric and family traits of children and their parents in ANCOVA. Residual height and cranial volume are in standard deviation units, i.e. age-specific values transformed to z-scores within sexes. Last three predictors are factors with two levels; other predictors are continuous. "Birth-parents together" (coded as 1) compares children living with both birth-parents against all other family types (single-parent and step-families coded as 0). "Father's education" is coded as 1 if the father had primary education and 0 if the father had secondary or tertiary education. "Meat shortage" is coded as 1 if the children reported meat shortage and 0 if they did not report it. *N* = 440 for height and 436 for cranial volume. Resource rating is given at 6-point scale (0 to 5). Units for continuous predictors are shown in the first column of Table 1. η^2 is a partial η^2, a measure of effect size (variance explained by a given variable of the variance remaining after excluding variance explained by other predictors). *β* is a standardised regression coefficient.

**A** Height of boys, R^2 = 0.32, *n* = 195				
Effect	*F*	η^2	*β* (*SE*)	*p*
**Mother's age at birth**	**8.6**	**0.045**	**0.19 (0.06)**	**0.00371**
**Mother's height**	**30.8**	**0.143**	**0.36 (0.06)**	**<0.00001**
**Father's height**	**7.4**	**0.038**	**0.18 (0.06)**	**0.00724**
**Birth weight**	**12.5**	**0.063**	**0.22 (0.06)**	**0.00051**
Number of siblings	0.1	0	-0.02 (0.06)	0.815
Resource rating	3.2	0.017	0.11 (0.06)	0.074
Two birth-parents	2.7	0.015	0.10 (0.06)	0.100
Father's education	4.2	0.022	-0.13 (0.06)	0.043
Meat shortage	0.3	0.001	-0.03 (0.06)	0.608

**B** Height of girls, R^2 = 0.39, *n* = 245				
**Mother's age at birth**	**11.5**	**0.047**	**0.19 (0.06)**	**0.00080**
**Mother's height**	**43.0**	**0.155**	**0.36 (0.06)**	**<0.00001**
**Father's height**	**47.1**	**0.167**	**0.38 (0.06)**	**<0.00001**
Birth weight	0	0	0.00 (0.05)	0.981
**Number of siblings**	**6.5**	**0.027**	**0.13 (0.05)**	**0.011**
Resource rating	2.5	0.010	0.09 (0.06)	0.116
Two birth-parents	0	0	0.01 (0.05)	0.830
Father's education	0.1	0	-0.02 (0.05)	0.733
Meat shortage	0.1	0	-0.01 (0.06)	0.801

**C** Cranial volume of boys, R^2 = 0.17, *n* = 195				
Effect	*F*	η^2	*β* (*SE*)	*p*
Mother's age at birth	2.5	0.013	0.11 (0.07)	0.117
Mother's height	0.4	0.002	0.04 (0.07)	0.538
**Father's height**	**6.5**	**0.034**	**0.18 (0.07)**	**0.012**
**Birth weight**	**13.6**	**0.069**	**0.26 (0.07)**	**0.0003**
Number of siblings	0.5	0.003	-0.05 (0.07)	0.482
Resource rating	1.4	0.007	0.08 (0.07)	0.241
Two birth-parents	1.2	0.006	0.07 (0.07)	0.281
Father's education	0.3	0.002	-0.08 (0.14)	0.569
Meat shortage	0	0	-0.00 (0.19)	0.993
Father's education* Meat shortage	0.2	0.001	0.09 (0.21)	0.677

**D** Cranial volume of girls, R^2 = 0.18, *n* = 241				
**Mother's age at birth**	**3.7**	**0.016**	**0.12 (0.06)**	**0.057**
Mother's height	0.5	0.002	0.04 (0.06)	0.488
**Father's height**	**6.9**	**0.029**	**0.17 (0.06)**	**0.009**
**Birth weight**	**6.3**	**0.027**	**0.16 (0.06)**	**0.012**
Number of siblings	0	0	0.00 (0.06)	0.987
**Resource rating**	**3.5**	**0.015**	**0.12 (0.07)**	**0.064**
Two birth-parents	2.4	0.010	0.10 (0.06)	0.121
**Father's education**	**13.0**	**0.054**	**-0.28 (0.08)**	**0.0004**
**Meat shortage**	**4.5**	**0.019**	**-0.19 (0.09)**	**0.034**
**Father's education* Meat shortage**	**6.9**	**0.029**	**0.26 (0.10)**	**0.009**
